# A Case of Perforated Duodenal Ulcer, with Remarks

**Published:** 1903-09

**Authors:** G. Munro Smith

**Affiliations:** Surgeon to the Bristol Royal Infirmary.


### A CASE OF PERFORATED DUODENAL ULCER, WITH REMARKS.

BY

G. Munro Smith,

*Surgeon to the Bristol Royal Infirmary.*

C. F., aged 33, was admitted to the Bristol Royal Infirmary at one o'clock a.m. on January 14th, 1903.

He had suffered for several years from abdominal pain, never very severe, usually between meals (not immediately after food), and during the night. The pain was ill-defined, but generally more on the right than the left side, and usually relieved by a little whisky. He has never had jaundice or urinary trouble, neither has he suffered from sickness, hæmatemesis or melæna.

He passed the day of January 13th in his accustomed health; he ate nothing out of the way except a little German sausage. As he was going upstairs to bed at eleven o'clock he was seized with a violent pain in the right side of the abdomen. This got worse, and he walked with difficulty to the Infirmary.

On admission he was moderately collapsed, contorted with pain, the abdomen was rigid and tender, pulse 84, respiration 20. The pain was paroxysmal, returning every few minutes.

There was no very obvious distension, and liver dulness was present. The most tender spot was over the region of the appendix.

At 2 a.m. he was put under ether, and I opened the abdomen in the middle line below the umbilicus. The peritoneal cavity contained turbid, yellowish green fluid without fæcal odour. The appendix was normal. I then opened above the umbilicus and explored the stomach. Here the evidences of peritonitis were more marked, and before long a circular ulcer, 3–5 mm. in diameter, was found on the anterior surface of the first part of the duodenum, about an inch from the pylorus. To obtain better access to the part the right rectus muscle was divided transversely. The ulcer was closed with silk sutures (Lembert) and the peritoneal cavity well cleansed with sponges through both incisions. A gauze drain was inserted into the upper and a rubber tube into the lower wound. The muscles were sewn up with catgut.

His condition during the day was favourable, but there was considerable pain, necessitating morphia injections. He was fed rectally.

On the 15th there was slight jaundice. He vomited dark blood occasionally; pulse improved.

On the 16th the jaundice was deeper. During the night he had violent hiccough; his pulse went up to 160 and he became collapsed, but soon rallied. The bowels were freely evacuated by a rectal wash.

After this he got on very well; the drains were removed on the 21st, the stitches on the 28th. He was allowed "albumin water" and peptonized milk by the mouth on the 16th, and minced chicken on the 29th.

On the 30th he had a sudden attack of very severe pain with alarming collapse; but this was soon relieved by a small hypodermic of morphia and hot fomentations.

His subsequent recovery was uneventful.

The first successful operation for perforated duodenal ulcer was, apparently, performed by Mr. Percy Dean in February, 1894.[1] Without operation the diagnosis is bound to be uncertain. The uncertainty lies between perforation of a gastric or duodenal ulcer, or appendicitis especially. Mr. Bolton[2] has pointed out the occasional close resemblance to appendicitis; and it is interesting that when the House Surgeon at the Infirmary summoned me to this case he remarked that he thought it was "appendicitis or duodenal ulcer."

Before perforation the diagnosis is also obscure; the main

[1] *Lancet*, 1894, i. 1191. [2] *Med. Rec.*, 1900, lvii. 494, 522.

points which indicate the duodenum rather than stomach are:—(1) Vomiting is not so common, neither is hæmatemesis; (2) duodenal ulcer is more common in males (3-1): gastric is much more frequent in females; (3) Tarry stools are more common; (4) The pain is apt to be in the right hypochondrium, an hour or two after meals (not soon after), and not immediately increased by food; (5) The average age for duodenal ulcer is 31.3 (average of 72 cases), and therefore rather later than the common age for gastric ulcer.

As to the operation. These ulcers are generally situated on the first part of the duodenum, and can generally be reached by a mediam incision; but it is a pity to waste time in attempts to draw up the gut, when a cut across the right rectus muscle makes it so much easier. In these and similar cases time is an important element in the operation: the chance of recovery is, I feel convinced, very much diminished by a long operation.

In the after treatment everything depends on careful nursing and feeding. The nourishment for the first few days should be given by the rectum. Sips of water may be taken to relieve thirst, but it should be remembered that thirst is allayed more by absorption from the large intestine than from the stomach.

When feeding by the mouth begins, "albumin water" (made by beating up the white of egg with water) should be the first given, then peptonized milk and gruel, followed at due intervals by pounded chicken, sago, barley water, milk puddings, etc. It should be particularly noted that a free secretion of gastric juice is not desirable, and therefore substances which excite this secretion should be carefully avoided, such as beef essences, gelatine, and all kinds of condiments and extractives. Probably no food is so dangerous in the early treatment after perforated gastric and duodenal ulcers as patent beef extracts and essences. Milk is one of the safest foods; but the toughness of the clot should be prevented by peptonization, or the addition of barley water, lime water, or soda water, etc. Fish is frequently given as a digestible and unirritating food; but it is certain that some kinds of fish stay in the stomach and duodenum for several hours, especially fat fish and cod. Whiting appears to leave the stomach the soonest.

